# Prekallikrein Deficiency Presenting as Recurrent Cerebrovascular Accident: Case Report and Review of the Literature

**DOI:** 10.1155/2012/723204

**Published:** 2012-08-16

**Authors:** Esteban Uribe Bojanini, Arturo Loaiza-Bonilla, Agustin Pimentel

**Affiliations:** Division of Hematology and Department of Medicine, Sylvester Comprehensive Cancer Center, University of Miami Miller School of Medicine, 1475 NW 12th Ave Suite 3300, Miami, FL 33136, USA

## Abstract

We report the case of a woman with history of hypertension and hyperlipidemia presenting with recurrent episodes consistent clinically with cerebrovascular accidents (CVA), and MRI changes suggestive of ischemia versus vasculitis as their cause. No anatomical neurological, rheumatic, cardioembolic, or arteriosclerotic etiologies could be determined by extensive workup. Incidentally, the patient was found to have prolonged activated Partial Thromboplastin Time (aPTT) and a normal Prothrombin Time (PT); further testing revealed a prekallikrein deficiency. Since no other cause for the CVAs was established, and other prothrombotic states were ruled out, it is proposed that they are clinical manifestations derived from the prekallikrein deficiency, which in a patient with known cardiovascular risk factors could lead to thrombotic complications such as stroke.

## 1. Case Presentation

A 32-year-old African American woman presented to the neurology clinic for followup, after having 4 episodes of cerebrovascular accidents (CVA) in the last 4 years. The cause for the CVA episodes had not been established despite extensive studies, including magnetic resonance imaging (MRI), echocardiogram with bubble study, CT angiography, carotid Doppler ultrasound, Holter monitoring, and after implementing secondary prevention measures with aspirin, statin, and blood pressure control. All of the episodes were associated with sudden left-sided weakness and dysarthria, lasting up to 2 hours in the first three episodes and 2 weeks in the last one, but they were self-limited, with complete resolution of symptoms afterwards. Due to the unclear diagnosis an MRI was ordered, which revealed high FLAIR and diffusion-weighted imaging (DWI) signal surrounding the frontal horns of the lateral ventricle; this was suggestive of an ischemic etiology versus vasculitis. The patient was admitted to the hospital for complete workup, seeking a definite diagnosis.

The patient had a prior medical history of hypertension treated with verapamil, and dyslipidemia treated with simvastatin, and she was taking aspirin 81 mg daily since her first episode of CVA. No history of oral or transdermal contraception. Review of systems revealed no history of bleeding or other thrombotic events in the past. She denied prolonged bleeding after dental procedures, menometrorrhagia, or pregnancy losses. No past surgical history. She denied alcohol, tobacco abuse, or illicit drug abuse. Her family and social history were noncontributory.

On admission, her physical examination showed a normotensive, afebrile patient, with no signs of bleeding or thrombosis, neurological symptoms, or focal deficits. Her initial complete blood count and comprehensive metabolic panel were revealed within normal limits.

Evaluation of coagulation times revealed a prolonged aPTT of 99.4 sec, with a normal PT of 11 sec. She was not on any anticoagulation therapy. Mixing studies were ordered. The aPTT was corrected to 30.1 sec after a 1 : 1 mix with normal plasma, indicating a coagulation factor deficiency and excluding the presence of an inhibitor of the intrinsic system. The activities of factors VIII, IX, XI, XII, and Von Willebrand factor (vWF) were in the normal range. Fibrinogen was normal and lupus anticoagulant assays were negative. Platelet function assays were normal. The abnormal aPTT shortened with prolonged incubation. Following these results, a deficit in the contact phase of the intrinsic coagulation pathway was suspected, more particularly a prekallikrein deficiency. A prekallikrein (PK) activity level was ordered, which revealed a 1% activity (on both clotting and amidolytic activity assays). PK antigen level was undetectable, which led to the final diagnosis of prekallikrein deficiency.

In order to rule out other potential, concomitant prothrombotic states, several tests were ordered, including Protein C, Protein S, Antithrombin III, Beta 2 Microglobulin, Anticardiolipin antibodies, lupus anticoagulant, CD55/CD59 per flow cytometry, factor V Leyden, Jak-2, and Prothrombin gene 202010 mutation analyses. All of these were unremarkable. Homocysteine levels were normal, and Sickle Cell Screen was also negative.

To rule out vasculitis or any other rheumatoid disease, a panel was ordered including antinuclear antibodies, anti-SSA, anti-SSB, anti-Smith, anti-RNP, antiscleroderma-70, antiribosomal antibody, antidouble-stranded DNA, antineutrophil cytoplasmic antibodies, rheumatoid factor, and complement C3 and C4, which were all normal.

A 12-lead electrocardiogram revealed normal sinus rhythm with no arrhythmias. Magnetic resonance carotid and vertebrobasilar angiogram revealed no arterial flow defects or atherosclerotic disease. The patient was not considered a candidate for thrombolysis or systemic anticoagulation due to the fact that it was not clear whether it was an acute or chronic event.

After completion of the aforementioned diagnostic workup, the patient was discharged home with diagnosis of prekallikrein deficiency. She was recommended to continue close surveillance by neurology and hematology, and to continue on daily aspirin and her hypertension and dyslipidemia medications.

## 2. Discussion

Prekallikrein (PK) is a glycoprotein synthesized in the liver and secreted as a single-chain peptide with a molecular weight of 88,000 Daltons and a normal plasma concentration of approximately 40 mg/mL [1, 2]. More than 75% of prekallikrein circulates as a complex with high-molecular-weight kininogen (HMWK). Only about 2–5% is free. PK together with Hageman factor (factor XII) and HMWK constitute the contact phase of the intrinsic coagulation and fibrinolytic pathways [3]. Its activation leads to the formation of factor XIIa, factor XIa, and kallikrein (KK) [4]. KK is implicated in many physiological and pathological processes including blood coagulation, the initiation of the classical complement cascade pathway, and the formation of bradykinin and renin [5].

Prekallikrein deficiency is a rare autosomal recessive disease, which was first described in 1965 by Hathaway et al. [6] in a family with 4 of 11 siblings showing a prolonged aPTT, caused by a deficiency of an unknown factor, which he called “Fletcher factor.” In 1973 Wuepper et al. [7] determined that the “Fletcher factor” was prekallikrein. Chromosomal localization of the gene has been mapped to the q34-q35 region on the long arm of chromosome 4, encoded by a single gene (*KLKB1*), and composed of 15 exons and 14 introns [8]. Seven types of mutations in prekallikrein deficient families have been identified by molecular genetic analysis, with most mutations being found in the light chain region of the prekallikrein gene [9].

Only around 80 cases of this disease have been reported in the literature, due to the fact that most of the patients are asymptomatic; it is likely that a large number of individuals go unrecognized and unreported [10]. Clinical manifestations are not clearly defined, as most of the patients remain asymptomatic and are diagnosed after an incidental finding of prolonged aPTT and a normal PT [11]. While PK, HMWK, and factor XII are required for a normal aPTT, they do not appear to be essential for normal coagulation. Despite the presence of a prolonged aPTT, patients with PK deficiency have no bleeding tendency, mostly due to the fact that proteins of the contact system are not key elements of the coagulation pathway, and they rather play a secondary role in the generation of thrombin. It has been reported that such factors deficiencies do not result in a marked bleeding diathesis, as most of the factor XI activation that is preferentially mediated via platelets and thrombin [11, 12].

Other coagulation factors deficiencies have been reported to present occasionally with thrombotic events [13]. These events have been reported in hemophilia and von Willebrand disease (VWD), but also in rare conditions like fibrinogen or factor VII defects [14]. Only patients deficient in factor II or factor X have not been reported with thrombosis [15]. It is still unclear whether defects in the contact phase components of blood coagulation, including PK—which are not associated with bleeding diathesis—represent a mild prothrombotic state or not.

Recent studies have suggested that factor XII deficiency is not a prothrombotic condition since most of the patients who were reported with thrombosis also had other acquired or congenital known risk factors [16, 17].

Several reports of thrombotic events in patients diagnosed with prekallikrein deficiency have been published. In 1983, Goodnough et al. [18] reviewed all 37 patients that had been diagnosed with PK deficiency and found 4 cases of thrombosis (10.8%). In 2010, Girolami et al. [10] reviewed the total of 75 patients reported with the deficiency, and found 9 thrombotic events (12.2%), which is a very similar incidence between the 2 reviews. Girolami et al. also reported associated prothrombotic risk factors in 6 of the 9 cases. They concluded that it is certain that PK deficiency does not protect from thrombosis in spite of the marked in vitro clotting defect, but a clear association with prothrombotic state cannot be made [10]. A limitation of these studies was that many patients were not studied for congenital prothrombotic defects.

## 3. Conclusion

In this paper we report the case of a woman with history of hypertension and hyperlipidemia presenting with recurrent episodes consistent clinically with cerebrovascular accidents (CVA), and MRI changes suggestive of ischemia versus vasculitis as their cause. It is relevant that the patient had previous diagnosis of hypertension and dyslipidemia, as they are known risk factors for stroke. No anatomical neurological, rheumatic, cardioembolic, or arteriosclerotic etiologies could be determined by extensive workup. Incidentally, the patient was found to have prolonged activated Partial Thromboplastin Time (aPTT) and a normal Prothrombin Time (PT); further testing revealed a prekallikrein deficiency. It is still unclear whether PK deficiency can be associated with prothrombotic state or not, and the rarity and underreporting of such cases make a prospective study a difficult task. In this particular case, since no other cause for the CVAs was established in our patient, and other prothrombotic states were ruled out, it is proposed that they are clinical manifestations derived from the prekallikrein deficiency, which in a patient with known cardiovascular risk factors could lead to thrombotic complications such as stroke. The infrequency with which PK is encountered and its pathophysiology make it a formidable diagnostic and therapeutic challenge.

## Supplementary Material

Coagulation cascade and relationship of PK in contact phase.
