# Supplementary material for: Prekallikrein Deficiency Presenting as Recurrent Cerebrovascular Accident: Case Report and Review of the Literature
Source: Case Rep Hematol. 2012 Aug 16;2012:723204. doi: 10.1155/2012/723204 (PMC3431062; doi:10.1155/2012/723204)
Supplement: Supplementary file 1 — Coagulation cascade and relationship of PK in contact phase. [file 723204.f1.pdf]

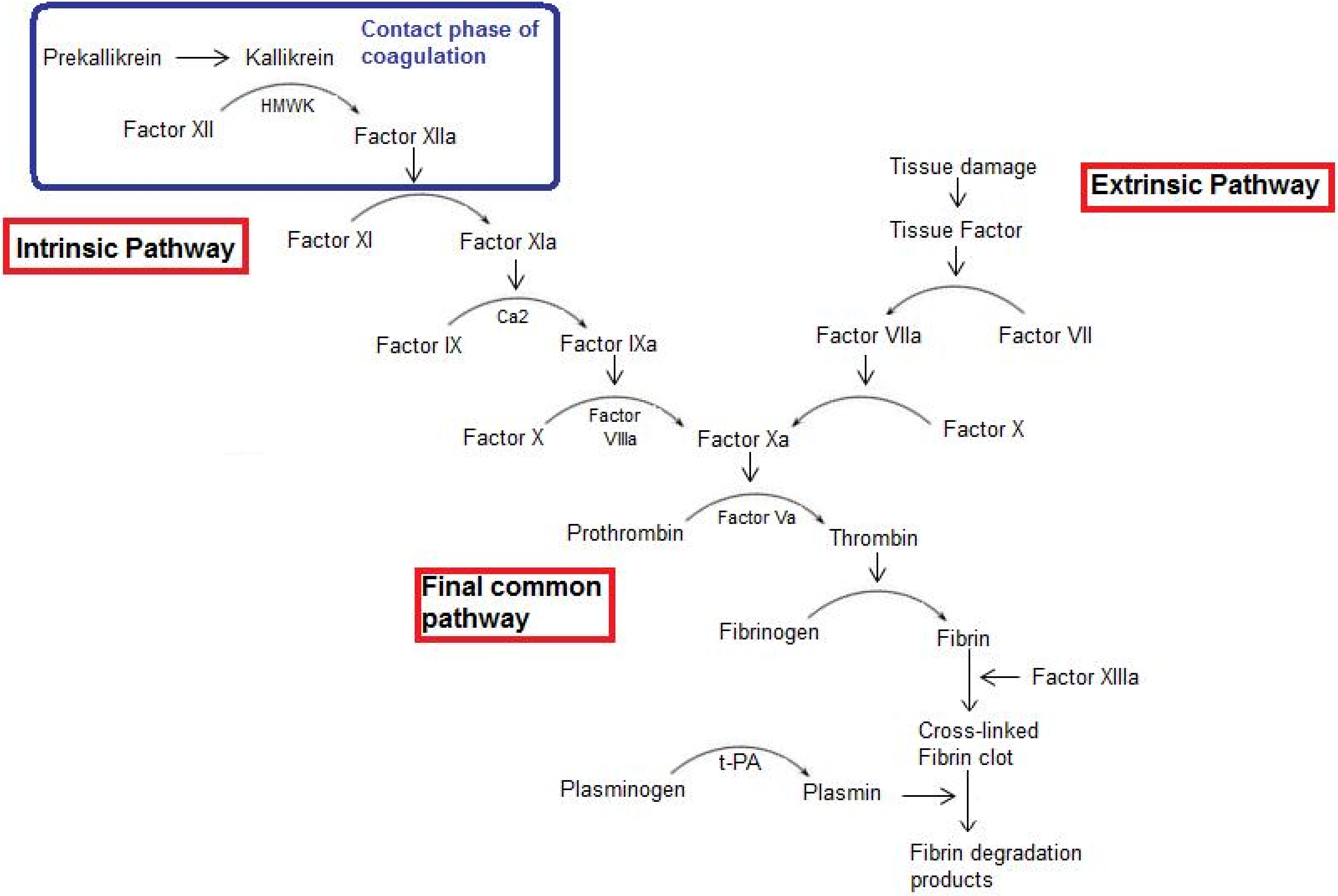
Contact phase of coagulation
Prekallikrein
Kallikrein
HMWK
Factor XII
Factor XIIa
Intrinsic Pathway
Factor XI
Factor XIa
Ca2
Factor IX
Factor IXa
Factor X
Factor VIIIa
Factor Xa
Tissue damage
Extrinsic Pathway
Tissue Factor
Factor VIIa
Factor VII
Factor X
Prothrombin
Factor Va
Thrombin
Final common pathway
Fibrinogen
Fibrin
Factor XIIIa
Cross-linked Fibrin clot
Plasminogen
t-PA
Plasmin
Fibrin degradation products
